# Influence of IFN-gamma and its receptors in human breast cancer

**DOI:** 10.1186/1471-2407-7-158

**Published:** 2007-08-14

**Authors:** Ignacio García-Tuñón, Mónica Ricote, Antonio Ruiz A, Benito Fraile, Ricardo Paniagua, Mar Royuela

**Affiliations:** 1 Department of Cell Biology and Genetics. University of Alcalá, E-28871. Alcalá de Henares, Madrid, Spain; 2 Department of Pathology, Hospital Príncipe de Asturias, E-28871 Alcalá de Henares, Madrid, Spain

## Abstract

**Background:**

Interferons are a group of proteins that trigger multiple responses including prevention of viral replication, inhibition of cell growth, and modulation of cell differentiation. In different mammary carcinoma cell lines IFNγ induces growth arrest at mid-G1. At the present there are no *in vivo *studies in human breast. The aim of this study was to investigate the expression patterns of IFNγ and its two receptors (IFNγ-Rα and IFNγ-Rβ) by Western blot and immunohistochemistry, in order to elucidate its role in the different types of human breast cancer (*in situ *and infiltrative).

**Methods:**

Immunohistochemical and semiquantitative study of IFNγ, its receptors types (IFNγ-Rα and IFNγ-Rβ), cell proliferation (proliferating cell nuclear antigen, also named PCNA), and apoptosis (TUNEL method) was carried between the three breast groups (fibrocystic lesions, *in situ* tumors and infiltrating tumors).

**Results:**

In the three groups of patients, IFNγ and IFNγ-Rα immunoreactions appeared in the cytoplasm while IFNγ-Rβ also was found in the nucleus. The optical density to IFNγ was higher in *in situ *carcinoma than in benign and infiltrating tumors. When we observed IFNγ-Rα, the optical density was lower in infiltrating carcinoma than in benign and *in situ *tumors (the higher density). To IFNγ-Rβ, the optical density was similar in the three group samples. In tumor samples PCNA and TUNEL index was significantly higher; than in benign diseases. PCNA index increased with the malignance. No significant differences were found between cancer types to TUNEL. IFNγ could be a potential therapeutic tool in breast cancer. However, tumor cells are able to escape from the control of this cytokine in the early tumor stages; this is probably due to a decreased expression of IFNγ, or also to an alteration of either its receptors or some transduction elements.

**Conclusion:**

We conclude that the decrease in the % positive samples that expressed IFNγ and IFNγ-Rα together with the nuclear localization of IFNγ-Rβ, could be a tumoral cell response, although perhaps insufficient to inhibit the uncontrolled cell proliferation. Perhaps, IFNγ might be unable to activate p21 to stop the cell cycle, suggesting a possible participation in breast cancer development.

## Background

Interferons belong to a protein family involved in viral replication prevention, cell growth inhibition and cell differentiation modulation [1]. IFNγ was described as a 17 kDa peptide that is secreted by antigen activated lymphocytes and natural killer cells. Also, it has been reported that IFNγ has antiviral activity, inhibits cell growth and modulates cell differentiation [2]. IFNγ acts throughout a specific membrane receptor, which is composed by two different subunits: IFNγ-Rα and IFNγ-Rβ [3]. IFNγ-Rα is sufficient for ligand binding but it is necessary the presence of Rβ to begin the IFNγ signal transduction [4]. IFNγ receptor complex has been described in a number of different tissues as endothelial cells, fibroblasts, neuronal cells, melanocytes and prostate cells [5,6].

In addition to its role in immune cells, IFNγ inhibits the growth of a number of nonhematopoietic cell types, including several tumor types. In fact, it has been considered as an antitumor agent [7-9].

The IFNγ ability to inhibit the growth of several tumor cell lines, including breast cancer cells, has been demonstrated in different studies [10-12]. This effect requires the signal transduction through the IFNγ receptor, so the tumor proliferation rate was higher in animal cells that expressed lower number of functional receptors [13,14].

In breast cancer patients with skin metastasis, local injection of IFNγ results in the total or partial regression of the skin lesions [15]. Other authors have been showed that IFNγ increases the growth inhibitory effect of tamoxifen in breast metastatic carcinomas [16,17]. In this way, it has been show that IFNγ produce this antitumoral effect up-regulating the expression of p21 and resulting cell cycle arrest in breast cancer cell lines [18].

At present and in our knowledge, there are no studies of IFNγ and its receptors expression reported by immunohistochemistry and western blot, in different non malignant and carcinomatous human breast tissue. The aim of this study was to elucidate the expression patterns of IFNγ and its receptors in human benign breast lesions and in *in situ *and infiltrating breast cancers, and to relate to the proliferation and apoptosis levels found in this tissues.

## Methods

### Patients and histological samples

Total or partial mastectomy specimens obtained from 52 women, who were clinically and histopathologically diagnosed of breast adenocarcinoma during 1998 in our hospital, were used for the study. Seventeen of these women (aged from 37 to 75 years; mean: 52.8) presented *in situ *carcinoma; one of these 17 women also showed lymph node infiltration at the time of surgery. Thirty-five women (aged from 40 to 82 years; mean: 59.94) had infiltrating carcinoma; 13 of these 35 women showed lymph node infiltration at the time of surgery, and 7 of these 13 developed metastasis 7–24 months after surgery. At present (January 2005), neither the remaining 22 women with infiltrating tumors nor the 17 women with *in situ *tumors have developed metastasis.

Tumor samples were compared with breast biopsies from 13 women (aged from 16 to 59 years; mean: 43.8) with benign lesions including ductal and lobular hyperplasia, fibroadenoma and fibrocystic changes. We always use the normal regions into these biopsies. All infiltrative tumor samples were classified by the TNM system (UICC, 1968).

Each sample was divided into two portions: one that was immediately processed for immunohistochemistry and the other were frozen in liquid nitrogen and maintained at -80°C for Western blot analysis. Removal of tissues was made by a Gynaecological Specialist MD and the samples were diagnosed by a Pathological Specialist MD (Ruiz A.). All pathological, clinical or personal data were anonymized and separated from any personal identifiers. This study was made with the consent of the written patients' relatives or their family in autopsy cases. All the procedures followed were examined and approved by the Principe de Asturias Hospital Ethics Committee and were in accordance with the ethical standards of the responsible committee on human experimentation, with the Helsinki Declaration of 1975 (revised in 1983) and the Committee on Publication Ethics (COPE) guidelines.

### Antibodies

The primary antibodies used were: mouse anti-human PCNA; goat anti-human IFNγ; and rabbit anti-human IFNγ-Rα and IFNγ-Rβ (Santa Cruz Biotechnology, California, Ca, USA). In Western blot analysis mouse anti-chicken α-actin (Amersham, Madrid, Spain) was also used to examine the relative expression of the other proteins.

### Western blot analysis

For Western blot analysis, tissues were homogenized in the extraction buffer (0.005 M Tris-HCl, pH 8) with addition of a cocktail of protease inhibitors (10 mM iodoacetamide, 100 mM phenylmethyl sulphonic fluoride, 0.01 mg/ml of soybean trypsin inhibitor and 1 μl/ml of leupeptin) and phosphates inhibitors (10 mM sodium fluoride and 1 mM sodium orthovanadate) in the presence of 0.5% Triton X-100. Homogenates were centrifugated for 10 min at 10,000 rpm. The protein concentration of supernatants was calculated by the Bradford method. Then, supernatants were equilibrated with loading buffer (10% SDS in Tris/HCl pH 8 containing 50% glycerol, 0.1 mM 2-beta-mercaptoethanol and 0.1% bromophenol blue) at 50 μg/ml. The mixture was denatured for 5 min at 100°C, and aliquots of 10 μl of homogenate were separated in SDS-polyacrylamide slab minigels (15% gels).

Separated proteins were transferred in the transfer buffer (25 mM Tris-HCl, 192 mM glycine, 0.1% SDS and 20% methanol). Nitrocellulose membranes (0.2 μm), were blocked for 1 h with 1% donkey serum in TBS, and incubated overnight at room temperature with the primary antibodies at 1:150 (IFNγ and IFNγ-Rβ), 1:500 (IFNγ-Rα) or 1:10000 (α-actin) in TBS with 5% bovine serum albumine (BSA). After extensive washing with TBS/Tween-20 (TBST), the membranes were incubated with donkey anti-goat (IFNγ) and swin anti-rabbit (IFNγ-Rα and IFNγ-Rβ) or rabbit anti-mouse (α-actin) biotinylated immunoglobulins (Dako, Barcelona, Spain) for 1 h at 1:2500 dilution in TBS with 5% BSA; and then washed and incubated with the avidin-biotin-peroxidase complex (Vector Laboratories, Burlingame, CA) at 1:1000 dilution. Furthermore, mouse anti-chicken α-actin (Amersham, Madrid, Spain) was also used at the same dilution to examine the relative expression of the other proteins. After an intensive wash, the filters were developed with an enhanced chemiluminescence (ECL) kit, following the procedure described by the manufacturer (Amersham, Buckinghamshire, UK).

### ELISA analysis

For ELISA procedure, the protein concentration of homogenates was calculated by the Bradford method. The different antigens were coated on 96 well multiplates overnight at 4°C. The plates were washed with TBS containing 0.05% Tween 20 and blocked with 1% BSA in TBS for 1 h at room temperature, and incubated with the different antibodies (anti-IFN-γ, IFNγ-Rα, IFNγ-Rβ) for 3 h also at room temperature. After a new wash, the biotin-conjugated anti-rabbit or anti-goat immunoglobulins (Dako) were added to each well, incubated for 1 h at room temperature, and then washed and incubated with the avidin-biotin-peroxidase complex (Vector). The interactions were visualized with 0.05% 2,2 azino di-3-etilbenziatioazolina sulphonic acid (ABTS) (Sigma) in 100 mM citrate buffer, and were measured (optical density at 405 nm) in a spectrophotometer (Multiskan Bichromatic, Labsystems, Finland).

### Immunohistochemical analysis (IHC)

Tissues were fixed for 24 h at room temperature in 0.1 M phosphate-buffered 10% formaldehyde, dehydrated and embedded in paraffin. Sections (5-μm thick) were processed following the avidin-biotin-peroxidase complex (ABC) method. Following deparaffinization, sections were hydrated, incubated for 30 min in 0.3% H_2_O_2 _diluted in methanol to reduce endogenous activity. To retrieve the antigen, the sections were incubated with 0.1 M citrate buffer (pH 6) for 2 min in a conventional pressure cooker. After rinsing in TBS buffer, the slides were incubated with normal donkey serum at 10% in TBS for 30 min to prevent non-specific binding of the first antibody. Thereafter, the primary antibodies were applied at a dilution of 1:25 (IFNγ and IFNγ-Rβ), 1:125 (IFNγ-Rα) or 1:500 (PCNA) in TBS at 37°C overnight. Afterwards, the sections were washed twice in TBS and then incubated with donkey anti-goat (IFNγ), mouse anti-rabbit (IFNγ-Rα and IFNγ-Rβ) or rabbit anti-mouse (PCNA) biotinylated immunoglobulins (Dako, Barcelona, Spain) at 1:500 in TBS. After 1 h of incubation with secondary antibody, the sections were incubated with a standard streptavidin-biotin-complex (Vector Laboratories, Burlingame, CA, USA) and developed with 3,3'-diaminobenzidine (DAB), using the glucose oxidase-DAB-nickel intensification method.

Immunochemical procedure specificity was checked using negative and positive controls. For negative controls of immunoreactions, tissues of each type were incubated with preimmune serum at the same immunoglobulin concentration used for each antibody (Santa Cruz Biotechnology). As positive controls, histologic sections (immunohistochemistry) of thymus samples were incubated with the same antibodies.

### Co-localization studies

Co-localization studies for IFNγ-IFNRα or IFNγ-IFNRβ was carried out in the same sections of each group. The procedure was similar than in simple immunostaining, but sections were first labelled with goat anti-human IFNγ and rabbit anti-human IFNγ-Rα or IFNγ-Rβ (Santa Cruz Biotechnology). In a second step sections were incubated for 1 h with donkey anti-goat (IFNγ) and mouse anti-rabbit (IFNγ-Rα and IFNγ-Rβ) biotilinated immunoglobulins (DAKO). In a third step, IFNγ was revealed using the glucose oxidase-DAB-nickel intensification method; and IFNγ-Rα or IFNγ-Rβ was revealed using the AP-red substrate (Zymed) After this, sections were mounted in Aquatex (Merck & Co., Inc, Whitethouse Station, NJ, USA)

### Tunel method

For histological evaluation of apoptosis, the sections were processed by terminal deoxynucleotidyl tranferase-mediated dUTP nick end-labeling (TUNEL) method, using a TdT FragEL-DNA fragmentation detection kit (Calbiochem-Oncogene, Cambridge, MA, USA), following manufacturer's instructions. Sections were further permeabilized by incubation with proteinase K (20 μg/ml) for 15 min, and then sections were exposed for 5 min to 3% H_2_O_2 _for endogenous peroxidase inhibition and incubated in terminal deoxynucleotidyl tranferase (TdT) (0.25 U/μl) labeling reaction mix for 1.5 hours at 37°C, washed in TBS, incubated with the avidin-biotin-peroxidase complex (Vector), developed with DAB (Sigma) and intensified by the glucose-oxidase method. Positive and negative controls were also performed, using the HeLa cells slides provided for such purpose in the Calbiochem kit and following manufacturer's instructions. For additional controls, sections of the three group samples were processed either without TdT enzyme in the labelling reaction mix, as negative controls, or pretreated with DNAse (1 μg/m1) for generation of the positive control slides.

### Quantitative analysis

A comparative histologic quantification of immunolabeling among the different groups of breast samples (*in situ *adenocarcinomas, infiltrating adenocarcinomas and benign lesions) was performed for each of the four antibodies. Of each sample (breast), six histologic sections were selected at random. In each section, the staining intensity (optic density) per unit surface area was measured with an automatic image analyzer (Motic Images Advanced version 3.2, Motic China Group Co., China) in 5 light microscopic fields per section, using the ×20 objective. For each positively immunostained section, one negative control section (the following in a series of consecutive sections) was also used, and the optic density of this control section was taken away from that of the stained section. Data are given as optic density per unit surface area (1 μm).

### Statistical methods

From the average values obtained for each breast, the means ± SD for each breast group were calculated. The number of sections examined was determined by successive approaches to obtain the minimum number required to reach the lowest SD. The statistical significance between means of the different breast group samples was assessed by the Fisher exact or χ^2 ^test at p ≤ 0.05, by multiple pairwise comparisons of all the values for each breast zone, for each specific antibody separately.

## Results

### Western Blot analysis

**Figure 1:**
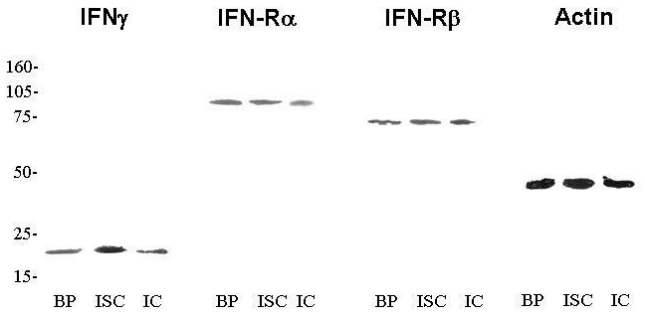
Western blot analysis of IFNγ, IFNγ-Rα, IFNγ-Rβ and α-actin after 15% polyacrilamide gel electrophoresis. BP, benign pathologies. ISC, *in situ *carcinoma. IC, infiltandrating carcinoma. For each antibody used, a single band – at their corresponding molecular weight – was found: IFNγ (17 kDa), IFNγ-Rα (80 kDa), IFNγ-Rβ (70 kDa) and α-actin (40 kDa).

For each antibody used, a single band – at their corresponding molecular weight – was found: IFNγ (17 kDa), IFNγ-Rα (80 kDa) and IFNγ-Rβ (70 kDa). The immunoreaction appeared in the three lesion groups (Fig. 1). Comparison of optical density band's revealed significant differences (p ≤ 0.05) between three breast groups: the highest optical density was found in *in situ *carcinomas (IFNγ and IFNγ-Rα); no significant differences were found to IFNγ-Rβ between the three sample groups (Table 1).

**Table 1:** Comparison of immunostaining intensities in Western blot analysis.

	IFNγ	IFNγ-Rα	IFNγ-Rβ	Actin
Benign lesions	9.75 ± 3.1^a^	18.59 ± 2.1^a^	18.81 ± 3.2^a^	57 ± 4.1^a^
*In situ *carcinoma	20.25 ± 1.9^b^	21.1 ± 3.6^a^	19.68 ± 4.1^a^	49 ± 3.4^a^
Infiltrating carcinoma	13 ± 2.7^a^	13.51 ± 2.2^b^	22.01 ± 6.8^a^	54 ± 3.8^a^

Comparison of immunostaining intensities (measured as average optical density ± SD) in Western blot analysis of bening breast lesions, *in situ *and infiltrating breast cancer. The labeled cells are classified in three groups according to their intensity range: low (< 10), intermediate (between 10 and 15) and high (>20). For each protein, values with different superscript letter differ significantly (P ≤ 0.05) between them. Those values sharing the same superscript are not statistically different from each other. Statistical analysis refers to each antibody separately. The mean values and SD for each group have been calculated from all samples corresponding to the group.

### ELISA analysis

**Figure 2:**
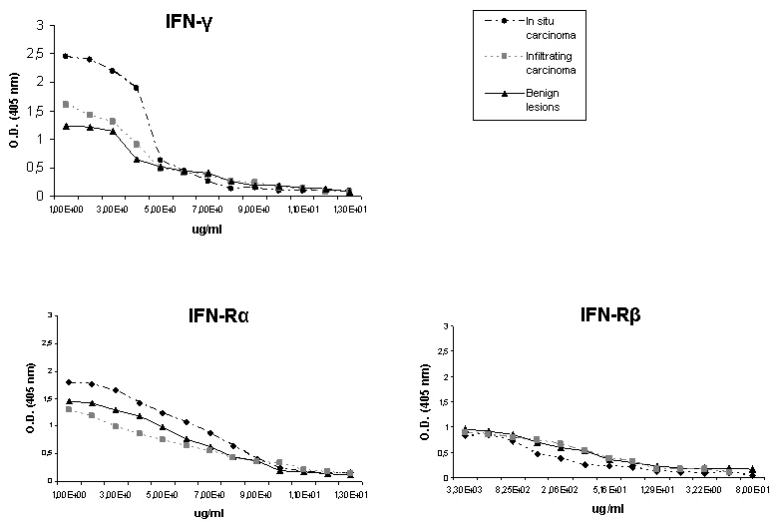
ELISA determinations of IFNγ and its receptors inbenign lesions, in situ carcinoma and infiltrating carcinoma. Antibody binding was followed at 405 nm, using peroxidase-conjugated immunoglobulins. The mean values and S.D. for each group have been calculated from five samples corresponding to the group. O.D., optical density.

In the ELISA analysis, a linear correlation was found between the optic density and the homogenized tissue increasing concentrations (Figs. 2).

### Immunohistochemistry analysis

No immunoreaction was observed in negative controls incubated with pre-immune serum, or using the antibodies pre-absorbed with excess of purified antigens. Immunoreaction to IFNγ and it receptor complex, always appeared in sections of human thymus (positive controls) for all antibodies (data not shown).

For the three proteins (IFNγ and it receptor complex), the percentage of positive samples (positive patient) and the optical density of immunoreaction are shown in Table 2.

**Table 2:** Comparison of immunostaining intensities and positive samples between groups of breast samples.

	IFNγ		IFNγ-Rα		IFNγ-Rβ		
	
	% positive samples	Optical density	% positive samples	Optical density	% positive samples		Optical density
						
					cytoplasm	nucleus	
Benign lesions	70,6%	11.4 ± 2.5^a^	88.2%	14.9 ± 2.3^a^	41.17%		19.4 ± 2.8^a^
					42.8%	57.14%	
*in situ *carcinoma	35%	30.7 ± 8.1^b^	41.4%	18.2 ± 2.7^a^	55.1%		17.6 ± 4.1^a^
					6.25%	93.75%	
Infiltrating carcinoma	40%	18.9 ± 2.1^a^	28.9%	9.9 ± 2.1^b^	22.2%		18.6 ± 2.9^a^
					20%	80%	

Comparison of immunostaining intensities and positive samples (obtained by inmunohistochemical analysis) between groups of breast samples. The labelled cells are classified in three groups according to their intensity range: low (< 10), intermediate (between 10 and 15) and high (>15). Mean ± SD. Values denoted by different superscripts are significantly different from each other. Those values sharing the same superscript are not statistically different from each other. Statistical analysis refers to each antibody separately. Significance was determined by multiple comparisons by the Fisher test at p ≤ 0.05.

In infiltrating tumor samples, imunoreactions to IFNγ, IFNγ-Rα and IFNγ-Rβ were compared with several tumor parameters (nodal status, TNM system and ER/PR status) (Table 3).

**Table 3:** Comparison of expression with clinical parameters.

	Number of cases (45)	IFNγ*	IFNγ-Rα*	IFNγ-Rβ*
Nodal status				
Negative	23	7	7	5
Positive	22	11 *p *= 0.416	6 *p *= 1.000	5 *p *= 1,000
TNM				
T1	17	7	8	6
T2	18	9	4	3
T3	6	1	1	1
T4	4	1 *p *= 0.756	0 *p *= 0.396	0 *p *= 0.543
ER				
Negative	6	5	0	4
Positive	39	13 *p *= 0.268	13 *p *= 0.320	6 *p *= 0.071
PR				
Negative	15	8	3	2
Positive	30	10 *p *= 0.563	10 *p *= 0.734	8 *p *= 0.706

Comparison of expression IFNγ, IFNγ-Rα and IFNγ-Rβ (only positive samples in each group were compared) with several anatomo-pathological features as nodal status, the TNM system and the ER/PR status in breast infiltrating carcinoma samples. *Number of positive cases

#### IFNγ immunostaining

**Figure 3:**
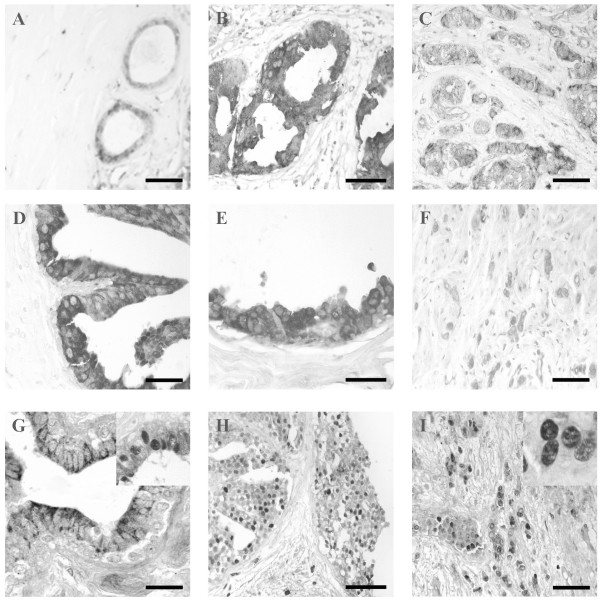
A-C. Immunostaining to IFNγ was observed always in the cytoplasm of epithelial cells. Similar immunoreaction was found in benign lesion (A) and in infiltrating carcinomas (C). Cases of *in situ *carcinoma (B) showed the highest immunoreaction intensity. Bar: 20 μm (A) and 15 μm (B and C). D-F: Immunostaining to IFNγ-Rα is localized in the cytoplasm of epithelial cells in the three breast groups. Similar immunoreaction was found in benign lesion (D) and in *in situ *patients (E). The reaction intensity was lower in infiltrating carcinomas (F) than in the other two groups. Bar: 15 μm (D and E) and 25 μm (F). G-I: The three sample groups showed a similar immunoreaction to IFNγ-Rβ. Immunostaining is localized in both the peripheral cytoplasm (G) and the nucleus (inset) in benign lesion group. This immunoreaction intensity was similar to that found in the other two groups. Most of *in situ *samples (H) and infiltrating carcinoma cases (I) showed nuclear immunostainig to IFN-Rβ. Bar: 10 μm (G) and 20 μm (H and I).

IFNγ was immunolocalized always in the cytoplasm of epithelial cells. The highest percentage of positive samples (70.6%) to IFNγ was found in the benign lesion group (Fig. 3A), while in tumor cases the number of positive samples was lower. The most intense immunoreaction was found in the 35% *in situ *tumors (Fig. 3B). The percentage of positive infiltrating tumors was similar as that found in *in situ *cases, but the intensity of immunoreaction was slightly decreased (Fig. 3C).

#### IFNγ-Rα immunostaining

The IFNγ receptor α was localized in the epithelial cell cytoplasm, in the three breast groups. The most of benign lesions (88.2%) were positive to Rα (Fig. 3D). Only 41.4% of *in situ *tumors were positive to α-chain receptor (Fig. 3E), but there were not significantly differences in the intensity of immunoreaction respect to that of the benign lesion group. A significantly decrease of immunoexpression and of percentage of positive samples (28.9%) were found in infiltrating breast tumors (Fig. 3F) in comparison with those of the other two groups (Table 2).

#### IFNγ-Rβ immunostaining

The three sample groups showed a similar immunoreaction to IFNγ-Rβ (Table 2). The IFNγ receptor β appeared in both cytoplasm and nuclei of epithelial cells of the three groups studied. A 41.1% of benign lesions was immunopositive to β-chain, in 42.8% the cytoplasm was positive (Fig. 3G), and 57.14% nucleus was positive (Fig. 3G inset).

A 55.1% of *in situ *tumors was positive to IFNγ-Rβ and in the most of them (93.76%) receptor β was immunolocalized in the nucleus (Fig. 3H).

In infiltrating carcinomas, only 22.2% of patients were positive to IFNγ-Rβ, but in 60% of positive samples IFNγ-Rβ appeared in the nuclei of epithelial cells (Fig. 3I inset).

#### Co-localization analysis

**Figure 4:**
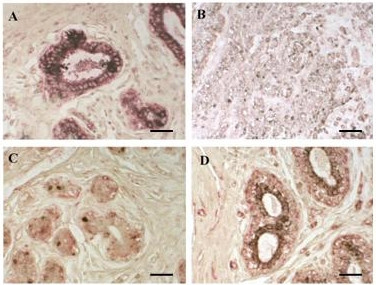
A-B Inmunostainig to IFN-γ (black) and IFN-Rα (red) in benign (A) and in infiltrating cancer (B). C-D Immunostainig to IFN-γ (black) and IFN-Rβ (red) in in situ (C) and in infiltrating breast cancer (D). Bar: 15 μm

The results obtained by immunohistochemistry were corroborated by co-localization analysis (Fig. 4).

### Proliferation and apoptosis analysis

The results of the immunoreaction to PCNA and TUNEL (expressed in positive cell percentages) are shown in Table 4. In benign diseases only 3.8% cells were positive to PCNA, while in tumor cases this percentage increased to 19% (*in situ*) and to 23.8% (infiltrating). The apoptosis index was 0.19% in the benign conditions. In tumor samples the index was significantly higher; no significant differences were found between cancer types.

**Table 4:** PCNA & TUNEL.

	**PCNA**	**TUNEL**
	
Benign lesions	3.8 ± 2.5^a^	0.19 ± 0.02^a^
*in situ *carcinoma	19.2 ± 5.5^b^	1 ± 0.4^b^
Infiltrating carcinoma	23.8 ± 5.6^c^	1.5 ± 0.5^b^

PCNA & TUNEL analysis were measured as average optical density ± SD. Values denoted by different superscripts are significantly different from each other. Those values sharing the same superscript are not statistically different from each other. Statistical analysis refers to each antibody separately. Significance was determined by multiple comparisons by the Fisher test at p ≤ 0.05.

## Discussion

Neoplasia occurs as a result of cellular changes that perturb normal balances between cell growth and cell death. These changes could be caused by an altered expression of several factors including IFNγ. In the present study we have found moderate expression of IFNγ (70.6% of cases) and IFNγ-Rα (88.2% of cases) in most of benign lesions samples. However, β subunit of this receptor appeared only in 41.2% of cases in this group, suggesting that there was a basal activity of IFNγ in non-neoplasic tissues. The IFNγ activity could be regulated by the expression of IFNγ-Rβ; therefore it is necessary to constitute the functional receptor to start the signal transduction [19]. Both IFNγ and its receptor appeared in the epithelial cells only, indicating that this cytokine acts in autocrine manner in order to control cell proliferation. Other authors stated that, in breast epithelial cells, the anti-proliferative effects of IFNγ occur by up-regulation of apoptotic members of the bcl-2 family [20]. In previous studies we reported low expression of bcl-2 in benign breast disorders [21]; this agrees with the low proliferation index and the high apoptotic index found in this group of cases. All these data are consistent with the proliferation/apoptosis equilibrium observed in non-malignant breast tissue.

In *in situ *carcinomas we found an increased expression of both IFNγ and IFNγ-Rα. However, the number of positive patients was lower than in benign diseases. There was no statistical difference in IFNγ-Rβ expression compared with benign diseases, but the location of this membrane receptor was in the nuclei of epithelial cells in most of *in situ *tumor cases (93.7%). These findings suggest a loss of IFNγ activity in breast epithelium in *in situ *tumors. Contrarily with our results, other authors have reported nuclear accumulation of IFNγ and IFNγ-Rα in the WISH cell line [22]; and, we found nuclear location only for IFNγ-Rβ. Doherty et al. [23] have stated that the lack of endogenous IFNγ at the tumor site, and also the inactivation of either the receptor components or the signal transduction pathway elements, could lead to a selective advantage for those mutated cells. In agreement with these statements, we have found a high proliferation index in the *in situ *tumor group. This could be caused by a lack of IFNγ activity due to the unusual location of β receptor subunit. This effect could mean the loss of the antitumoral effects of IFNγ in early stages of human breast cancer. Although this is the first time that IFNγ-Rβ was found in the nucleus. So other proteins such as p53 or p21 changed his location in breast cancer, probably due to mutant forms of these proteins that present different functions, as occurs in the most of cancers, and it could be associated with the cancer development and progression [24,25].

In prostate cancer cells it has been reported that IFNγ apoptotic effects are promoted by p21 stimulation, which inhibits G_1 _and S phase of cell cycle [26]. In breast cancer cell lines, IFNγ treatment produces an increase in p21 [18]. Our results suggest that IFNγ could be non-functional and unable to activate p21 to stop the cell cycle. In this sense, in a previous study, we detected p21 in the cytoplasm of epithelial cells in *in situ *carcinomas, and this suggests that the p21 cytoplasmic product does not reach to enter the nucleus, and therefore, it would not be functional and would not contribute to the cell cycle arrest [27]. Furthermore, this p21 location has been related to breast cancer resistance to the TNF-α apoptotic effect [28], and it could be explained by the IFNγ effects mediated by TNFRI [29]. According with these data, in a previous study we described a high expression of TNFRI in *in situ *carcinomas of the breast [30]. Although IFNγ effects mediated by TNFRI would be inhibited by the presence of p21 in the cell cytoplasm, the p21 location in *in situ *carcinomas would be capable to inhibit the apoptotic pathway of TNFRI at ASK-1 level [31], and thus, to prevent the IFNγ function as cell cycle inhibitor.

The group of infiltrating adenocarcinomas showed a lower expression of IFNγ than the *in situ *group, and a similar expression to that observed in the benign disease group. However, the number of positive cases was lower (40%) and similar to *in situ *carcinoma (35%). For IFNγ-Rα, the intensity of reaction and the number of positive cases decreased. IFNγ-Rβ showed similar reaction intensity in the two carcinoma groups, and the immunoreaction appeared in the cell nuclei. These data suggest a loss of IFNγ antitumoral activity in these tumors. IFNγ, via IRF-5 (*interferon regulatory factor-5*), can stimulate the expression of apoptotic genes as bax [32]. Therefore, the decreased IFNγ positive samples could result in an altered bax/bcl-2 balance. Ruiz-Ruiz and Lopez-Rivas [33] have suggested that, in breast cancer cell lines, IFNγ favors activation of mitochondria-operated apoptotic pathway by TRAIL; this activation can be inhibited in bcl-2 overexpressing cells. We reported an overexpression of bcl-2 in a previous study [21] in all these tumors and in the same patients used in this study; this overexpression could lead to the inhibition of IFNγ apoptotic effects, resulting in deregulation of the proliferation/apoptosis equilibrium towards proliferation. This effect could be the cause of the higher proliferation index found in these samples (up to 23% cells) respect to that observed in both benign diseases and *in situ *tumors. Therefore, present observations could suggest that IFNγ could be a potential therapeutic tool in breast cancer. However, tumor cells are able to escape from the control of this cytokine in the early tumor stages; this could be due to a decreased expression of IFNγ, or also to an alteration of either its receptors.

## Conclusion

We conclude that the decrease in the % positive samples that expressed IFNγ and IFNγ-Rα together with the nuclear localization of IFNγ-Rβ, could be a tumoral cell response, although possibly insufficient to inhibit the uncontrolled cell proliferation. IFNγ might be unable to activate p21 to stop the cell cycle, suggesting a possible participation in breast cancer development.

## Abbreviations

IFNγ : Interferon-γ; IFNγ-R: Interferon-γ receptor; PCNA: proliferating cell nuclear antigen; kDa: Kilodalton; TNM: tumor, nodes, metastasis; UICC: International Union Against Cancer; MD: Medical doctor; COPE: Committee on Publication Ethics; M: Molar; mM: MiliMolar; SDS: sodium dodecyl sulphate; TBS: Tris Buffered Saline ;BSA: bovine serum albumine; TBST: TBS/Tween-20; IHC: Immunohistochemistry; ABC: avidin-biotin-peroxidase complex; DAB: 3,3'-diaminobenzidine; TUNEL: terminal deoxynucleotidyl tranferase-mediated dUTP nick end-labeling method; TdT: terminal deoxynucleotidyl tranferase; SD: standard deviation; ER: Estrogen Receptor; PR: Progesterone receptor; TNF: Tumor necrosis factor; TNFR: Tumor necrosis factor receptor; ASK-1: apoptosis signal-regulating kinase 1 ; IRF-5: interferon regulatory factor-5; TRAIL: TNF-related apoptosis-inducing ligand.

## Competing interests

The authors declare that they have no competing interests.

## Authors' contributions

IG and MaR designed the study, carried out the immunohistochemistry studies, the Quantitative analysis and participated in discussion. MoR and RP participated in western blot analysis, result interpretation and discussion. AR prepared and provided the tumor biological samples and participated in the immunohistochemistry studies. BF performed the statistical analysis and participated discussion. MaR and RP participated in study coordination and supervision. All authors read, discussed and approved the final manuscript.

## Pre-publication history

The pre-publication history for this paper can be accessed here:


